# Stabilising CO_2_ concentration as a channel for global disaster risk mitigation

**DOI:** 10.1038/s41598-024-79437-5

**Published:** 2024-11-24

**Authors:** Saite Lu, Demosthenes Tambakis

**Affiliations:** 1 Emmanuel College, University of Cambridge, Cambridge, UK; 2 Pembroke College, University of Cambridge, Cambridge, UK

**Keywords:** Climate change mitigation, $\hbox {CO}_2$ concentration, Disaster incidence rate, Logit regression, Natural hazards, Environmental social sciences

## Abstract

We investigate the influence of anthropogenic $\hbox {CO}_2$ concentration fluctuations on the likelihood of climate-related disasters. We calibrate annual incidence rates against global disasters and $\hbox {CO}_2$ growth spanning from 1960 to 2022 based on a dynamic panel logit model. We also study the sensitivity of disaster incidence to stochastic carbon dynamics consistent with IPCC-projected climate outcomes for 2100. The key insight is that present and lagged $\hbox {CO}_2$ growth contains valuable information about the likelihood of future disaster events. We further show that lowering carbon stock uncertainty by dampening the persistence or the variability of $\hbox {CO}_2$ concentration has a first-order impact on mitigating expected disaster risk.

## Introduction

There is clear evidence that the annual incidence of climate-related disaster events, including droughts, floods, storms, heatwaves, and forest fires has risen consistently since the mid-20th century^1–22^. The positive trend is reflected in greater disaster severity, proxied by the number of affected individuals^23^, and negative impacts on output^8^ and productivity^24^. Further, it is very likely that there are tipping points in the Earth System—abrupt regime shifts to the temperature feedback from emissions driving global warming and to the deep oceans’ carbon sink capacity^1–9,25–27^—such that short-term disaster risk may coincide with collapsing biospheres and ecosystems, amplifying economic damage and human losses^6^. Climate science underscores the unequivocal anthropogenic influence of greenhouse gases-predominantly carbon dioxide ($\hbox {CO}_2$)-on disaster risk^28^. (The main non-$\hbox {CO}_2$ greenhouse gases (GHG), methane ($\hbox {CH}_4$) and nitrous oxide ($\hbox {N}_2$O) are also projected to grow in the 21st century^1^. $\hbox {CH}_4$ dynamics are particularly sensitive to global surface temperature tipping points. In this paper we abstract from their contribution to disaster risk. Reference^16^ studies the slow decay rate of GHG concentration levels: future warming due to the long time scales of ocean-linked heat transfer could remain for millennia even if human emissions were to stop immediately.)

This paper explores the predictive power of stochastic carbon stock dynamics for short-term disaster risk by modelling the sensitivity of 1-year-ahead disaster frequency to fluctuations in global average atmospheric $\hbox {CO}_2$ concentration. To this end, we expand upon the logit-based framework of Refs.^14,17^ for the likelihood of financial crises. Our analysis incorporates the annual global disaster record spanning 63 years (1960–2022) maintained by the Centre for Research on the Epidemiology of Disasters (CRED). Here, incidence denotes the annual conditional likelihood of a climate-related disaster event. Conditional disaster incidence is driven by the growth of carbon stock and adjusted for geographic factors including countries’ absolute distance from the equator, the length of their coastlines, and countries’ urban population share as a socio-economic control variable^27^. Based on the logistic elasticity estimates, the binary classification power (disaster/no disaster) of our model is evaluated using the Area Under the Receiver Operating Characteristic (AUROC) curve, which exceeds 85%. Further, the cumulative impact of 1% lower $\hbox {CO}_2$ concentration on certainty-equivalent (“no uncertainty”) disaster likelihood lies between $\sim 0.7$ and 1.1%. Such estimates are consistent with the short-term attribution of climate-related disasters to anthropogenic carbon emissions, documented in Ref.^29^ and quantified by Ref.^10,30^.

The paper’s contribution is twofold. First, it contributes to applied research by modeling the risk reduction potential of different decarbonisation strategies^19^. The research closest to our specification is^27^. These authors’ econometric method differs from ours in that they regress carbon emission levels to apply linear cointegration techniques, while we estimate a logit model. Our logistic approach is novel in a climate-change context because it captures the potentially nonlinear (sigmoid) relationship between disaster risk and carbon emissions. (The usefulness of the logistic approach was validated in economics literature by Ref.^14^, who demonstrated a nonlinear historical relationship between credit growth and the risk of financial crises, as well as in Refs.^31,32^. Another study, employing linear panel regressions across seven world regions, is^4^. Non-regression-based studies linking disaster risk to $\hbox {CO}_2$ emissions include^33^, using a dynamic stochastic general equilibrium (DSGE) model, and Ref.^34^, applying non-parametric machine learning.) Further, Ref.^27^ finds the elasticity of disasters to $\hbox {CO}_2$ emissions is $\sim 9$, implying a disaster count doubling-time estimate of 13 years. Against that, our estimated elasticities imply that under the current trend in atmospheric $\hbox {CO}_2$ concentration, the total disaster count would double from an average 1.7 to $\sim 3.4$ events per country-year over the next 15 to 25 years. This timeframe is broadly consistent with the 40% increase in global disasters by 2030 forecast by Ref.^21^ and with the shorter time window available for recovery following successive disaster events^22^. Disparities in doubling times are important for national and global stakeholders to judge the scale of disaster adaptation and mitigation policies. Lastly, Ref.^27^ controls for multiple socio-economic determinants but its scope is confined to hydrological disasters. By contrast, by including all climatological disasters events between 1960 and 2022 we obtain a more comprehensive measure of global climate-related disaster risk. While in 2000–2019 climatological disasters accounted for about 16% of all disaster events, unchanged from 1980 to 1999, extreme temperature events have more than tripled, from 130 to 432, and wildfires have also risen sharply^23^. In recent years more frequent and longer heat waves and droughts have drawn wide international media coverage.

Second, our reduced-form approach complements the fast growing research on the impact of climate risks on optimal climate policy. The results reinforce those arguing for a higher precautionary and insurance response to the volatility of carbon dynamics^35^. Because mean surface temperature sensitivity is concave (logarithmic) in the doubling of $\hbox {CO}_2$ stocks (Arrhenius’ Law^20^) the Social Cost of Carbon (SCC) impact of uncertainty about carbon flows is dampened, while that of uncertainty about temperature feedbacks is not. Such uncertainty may lead to the 200-year SCC rising by over 20%^36^. (The SCC represents the expected present discounted value of all future marginal damages resulting from emitting a ton of $\hbox {CO}_2$ today. When one ton of $\hbox {CO}_2$ is emitted into the atmosphere, approximately 0.45 tonnes remain, with the remainder being absorbed by land and ocean sinks. Although this atmospheric increase may exhibit significant short- and long-term variation due to feedback effects and tipping points, we can assume the conversion factor is constant as a first-order approximation. We are grateful to Glen Peters at the Center for International Climate Research (CICERO), Norway,  for this observation.) We find that scaling down the persistence and volatility of fluctuation has first-order effects on expected disaster risk. Climate science is advising that delaying mitigation efforts risks augmenting total disaster risk, with the associated short- and long-term costs^29^. Against this IPCC-led consensus, our key policy insight is that stabilizing carbon stock is an independent avenue for disaster risk reduction which acts to enhance any existing mitigation targets.

We run logit regressions with one-way (country) and two-way (adding time) fixed effects. The logistic specification is intuitive and allows for directly testing the underlying nonlinear relationship between $\hbox {CO}_2$ growth and one-period-ahead conditional disaster risk. The key role of nonlinearity is emphasised by the cumulative nature of disaster tipping points^9^ and the increasing interconnectedness of climate hazards^6^. We include lagged disaster risk on the right-hand side (see also Refs.^4,37^), but to minimise the risk of overfitting, we do not include interactive fixed effects (The results remain robust once interactive fixed effects are introduced. See Appendix B.), as suggested by Ref.^38^ in the baseline analysis. Other logistic applications to disaster risk exposure are mostly limited to a single sector or country, such as water resource management in South Korea^39^. To the best of our knowledge, our specification is the first to model the climate-induced disaster impact of carbon emissions on a global scale.

We find the certainty-equivalent disaster incidence rate is 6.2% annually. Separately, we estimate a stationary AR(3) process for atmospheric $\hbox {CO}_2$ concentration. We detect strong (3-year) persistence of disaster likelihood to historical $\hbox {CO}_2$ growth. The logit model is calibrated to carbon flows’ historical mean and standard deviation. Given the logistic transfer parameter estimates, we simulate Gaussian $\hbox {CO}_2$ growth shocks and generate 1-year-ahead disaster incidence trajectories controlling for countries’ urban population share. The lowest predicted $95\%$ confidence interval for stochastic incidence across our specifications is [6.7, 7.8]% p.a., and around $20\%$ of total disaster risk is attributed to carbon stock uncertainty. We also find disaster risk estimates and implied elasticities are greater with time-fixed indicator variables, reflecting the higher convexity of the logit parameters under two-way fixed effects. Although they are robust to parameter uncertainty, a caveat of these baseline estimates is our assumption of reliable vulnerability data, represented here by countries’ urban population share although disaster exposure is a multifaceted process^40^.

For sensitivity analysis we focus on the logit parameters estimated with one-way fixed effects. We calibrate the $\hbox {CO}_2$ growth mean to the end-horizon (2100) $\hbox {CO}_2$ stock level aligned with a specific IPCC Representative Concentration Pathway (RCP^2,41^), while $\hbox {CO}_2$ growth variability is either set to its historical baseline or adjusted to half the baseline value. Each pathway specifies a time series of future $\hbox {CO}_2$ and GHG concentrations leading to 2100. We find that certainty-equivalent disaster risk drops from $\sim 5.5$ to 1% p.a. along with average $\hbox {CO}_2$ concentration, while total disaster risk is reduced from $\sim 6.5$ to $\sim 1.2$% p.a. under baseline variability, with a corresponding narrowing of the uncertainty wedge. Successive negative increments to expected disaster risk exceed the continuous cumulative elasticities discussed above. Further, average risk reduction is greater (down to $\sim 1$% p.a.) if $\hbox {CO}_2$ concentration becomes more stable. This finding is supported by numerical evidence of the uncertainty wedge narrows to nearly zero. For expected disaster risk to fall, $\hbox {CO}_2$ concentrations must move away from its baseline mean and/or standard deviation. Nonetheless, we find the projected uncertainty wedge under the most pessimistic pathway for 2100 (RCP8.5, representing business-as-usual emissions rising throughout the 21st century) is just $\sim 0.1$% p.a. below baseline. By contrast, for the more ambitious RCP scenarios (the very stringent mitigation pathway consistent with RCP2.6, as well as the intermediate mitigation efforts associated with RCP4.5) maintaining the post-1960 average $\hbox {CO}_2$ growth rate while halving its variability would result in a lower total disaster risk compared to baseline variability and a near-zero ($<0.5$% p.a.) uncertainty wedge.

The key policy implication is that mitigation policies targeting lower average $\hbox {CO}_2$ concentration could achieve a greater expected reduction in disaster incidence if they also aimed to stabilise carbon growth. This paper argues that aggressive decarbonisation strategies not only offer the benefits of mitigating *short-term* (annual) disaster risks, but also contribute to dampening the catastrophic *long-term* (millennial) consequences for human civilisation if business-as-usual emissions continue, as this would very likely trigger multiple irreversible climate tipping points^9,13^. (Critical systems that we rely on for survival-including cryosphere subsystems (the Greenland and West Antarctic ice sheets), biosphere subsystems (the Amazon Rainforest), and large-scale oceanic and atmospheric circulation patterns such as the Atlantic Meridional Overturning Circulation (AMOC)-are at risk of collapse due to climate tipping points^5^. This would be disastrous for human society and the economy unless drastic carbon emission reductions are urgently implemented.) In a companion paper we study the long-term implications of these insights using non-homogenous Markov chains^18^.

In the remainder of the paper, “Data and methods” presents the data, defines disaster incidence, and derives the uncertainty wedge; “Results” outlines the empirical results; “Sensitivity analysis and discussion” contains sensitivity analysis; and “Concluding remarks” concludes. Appendix A describes the simulation methods and RCP assumptions, while Appendix B provides results from multiple robustness checks. Throughout, we refer interchangeably to disaster incidence and risk, as well as to $\hbox {CO}_2$ stock and concentration.

## Data and methods

### The data

We employ EM-DAT, the CRED database, for the occurrence and socio-economic impact of climate-related disaster events globally^23,42^. The primary hazards are hydro-meteorological—rainfall-induced flooding, related landslides, tropical cyclones and storms—and climatological, including droughts, wildfires, heatwaves and extreme cold events. Our analysis encompasses these extreme event categories delineated by country and year and recorded from 1959 to 2022. The total number of countries and territories ranges from 158 to 218 depending on the fixed effect specification. We exclude non-climate related geophysical (earthquakes and volcanoes) and biological events (virus outbreaks and epidemics) and include concurrent and multiple disaster events even if they overlap chronologically^28^. The incidence rate serving as dependent variable quantifies the conditional probability of a disaster event occurring in a specific country within a year. It is thus a disaster count unadjusted for the event’s duration.

We recognise that the EM-DAT maintained by CRED has several limitations that may impact the accuracy of our analysis. The dataset often lacks granularity, particularly for disasters that span multiple countries and years, making it difficult to accurately capture localized impacts. Additionally, EM-DAT includes only events meeting specific thresholds, which may lead to underreporting of smaller-scale disasters, particularly in countries with effective mitigation systems. There is also a lack of consistent reporting standards across countries, with better-resourced nations providing more comprehensive records compared to those with limited administrative capacity. Moreover, data gaps and inconsistencies in historical records affect comparability over time and between countries. The data on atmospheric $\hbox {CO}_2$ concentration is documented by the US National Oceanic and Atmospheric Administration (NOAA), measured in parts per million by volume (ppm) and adjusted for seasonality. (The growth in atmospheric $\hbox {CO}_2$ concentration is an underestimate of total GHG growth; (The main non-$\hbox {CO}_2$ greenhouse gases (GHG), methane ($\hbox {CH}_4$) and nitrous oxide ($\hbox {N}_2$O) are also projected to grow in the 21st century^1^. $\hbox {CH}_4$ dynamics are particularly sensitive to global surface temperature tipping points. In this paper we abstract from their contribution to disaster risk. Reference^16^ studies the slow decay rate of GHG concentration levels: future warming due to the long time scales of ocean-linked heat transfer could remain for millennia even if human emissions were to stop immediately.). NOAA initiated monthly $\hbox {CH}_4$ and $\hbox {N}_2$O concentration measurements in 1983 and 2001, respectively. The estimated uncertainty in the annual $\hbox {CO}_2$ mean is $\approx 0.12$ppm, derived from the annual mean values recorded by NOAA.)

**Fig. 1:**
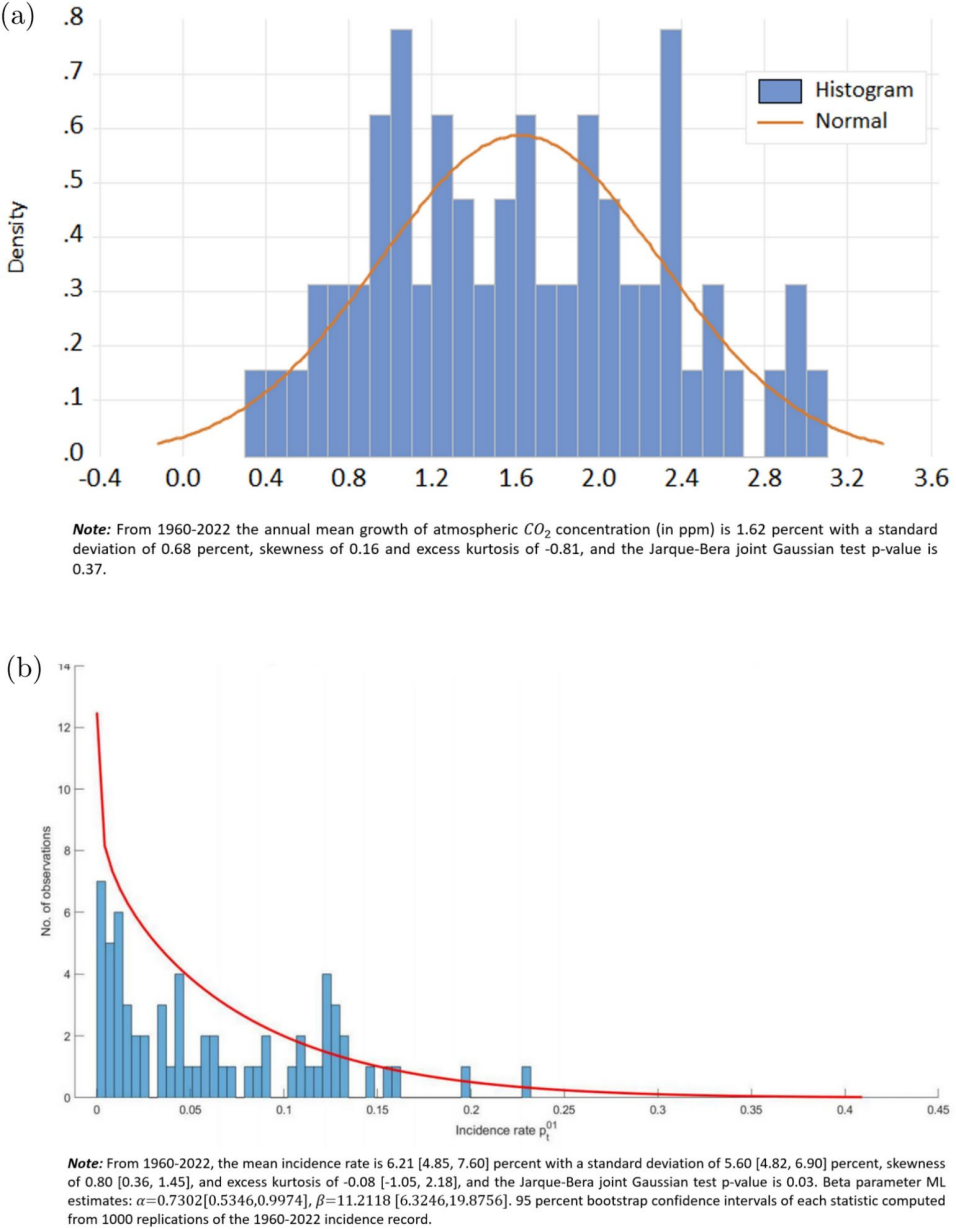
Descriptive statistics.

Figure 1 displays the empirical histograms of annual $\hbox {CO}_2$ growth rates in percent p.a. (panel (a)) and disaster incidence rates (panel (b)), superimposing smooth Gaussian and beta pdf’s fitted by maximum likelihood.

The mean incidence rate over the entire period (1960–2022) is 6.2%, with a standard deviation of 5.6% annually. Average incidence has been on an upward trajectory for shorter subperiods and its standard deviation has been declining (not reported).

### Disaster incidence and uncertainty wedge

Disaster incidence in year $t+1$, $p_{t+1}$, is the conditional probability of a disaster event occurring in year $t+1$ (binary state $s_{t+1}=1$) given no disaster in year *t* (binary state $s_t=0$). Disaster risk is often assumed to follow a Poisson arrival process in continuous time, e.g.^33^. By contrast, we define $p_{t+1}$ to be a logistic function of the growth in global average atmospheric $\hbox {CO}_2$ concentration in year $t-j$, $c_{t-j} {\mathop {=}\limits ^\textrm{def}} \Delta \log C_{t-j}$, with $j = 0,...,P$, and constant unconditional moments $E[c_{t-j}] = \mu _c$ and $var[c_{t-j}] = \sigma ^2_c$, all *j* by stationarity, and **X**$_t=[X_{1t},...,X_{Kt}]$ to be a $K \times 1$ vector of other relevant year-*t* information:

1 $$\begin{aligned} p_{t+1}&{\mathop {=}\limits ^\textrm{def}} p(c_t , {\textbf {X}}_t) = \Pr \{ s_{t+1}=1 \vert s_t=0 , X_{kt} \} \nonumber \\&= \frac{\exp \left( \overline{h} + \sum _{j=0}^P h_j c_{t-j} +\sum _{k=1}^K \gamma _k X_{kt} \right) }{1+ \exp \left( \overline{h} + \sum _{j=0}^P h_j c_{t-j} + \sum _{k=1}^K \gamma _k X_{kt}\right) } \end{aligned}$$

where $h_j$ and $\gamma _k$ are the elasticities of $p_{t+1}$ to $\hbox {CO}_2$ concentration growth in lag year *j* and non-carbon factor $k \in \{1,..., K\}$, respectively, and $\overline{h}$ is a scaling constant such that disaster incidence is bounded ($p_{t+1} \in [0,1]$). Evaluating $p_{t+1}$ at $\mu _{c}$ and $E[{\textbf {X}}_t]$ yields the certainty-equivalent disaster incidence rate, denoted $\overline{\mu }_p$:

2 $$\begin{aligned} \overline{\mu }_p {\mathop {=}\limits ^{\textrm{def}}} p(\mu _c, E[{\textbf {X}}_t] ) = \frac{\exp \left( \overline{h} + \sum _{j=0}^P h_j \mu _c +\sum _{k=1}^K \gamma _k \mu _k \right) }{1+ \exp \left( \overline{h} + \sum _{j=0}^P h_j \mu _c + +\sum _{k=1}^K \gamma _k \mu _k\right) } \end{aligned}$$

We note the average growth of $\hbox {CO}_2$ concentration, $\mu _c \approx 1.6$% p.a. from Fig. 1a impacts $\overline{\mu }_p$ while its volatility $\sigma _c \approx 0.7$% p.a. does not. Further, $p_{t+1}(\cdot )$ is strictly convex to the left of its unique inflexion point, hence the expected incidence rate in this range exceeds $\overline{\mu }_p$ by Jensen’s inequality. We henceforth refer to this analytical property of convex transfer as Property 1:

3 $$\begin{aligned} UW_{p} {\mathop {=}\limits ^{\textrm{def}}} E[p_{t+1}] - \overline{\mu }_p \ > \ 0 \end{aligned}$$

$UW_{p}$ is the positive uncertainty wedge between the two measures. In other words, Property 1 states that stochastic disaster risk exceeds its certainty equivalent when model-implied incidence is a convex function of annual mean $\hbox {CO}_2$ growth. Further, subject to scaling and translation, the logistic (sigmoid) function is proportional to a hyperbolic tangent function^3^:

4 $$\begin{aligned} UW_{p}(x) \propto \frac{e^x}{1+ e^x} - \frac{1}{2} = \frac{1}{2} \tanh (x/2) \end{aligned}$$

where *x* is the exponential argument in Eq. (1), $\overline{\mu }_p$ is scaled to $\frac{1}{2}$ wlog, and

5 $$\begin{aligned} \tanh (x/2) = x/2 - \frac{(x/2)^3}{3} + \frac{2 (x/2)^5}{15} - \frac{17 (x/2)^3}{315} + \cdots \, \ \ |x| < \frac{\pi }{2} \end{aligned}$$

From Eqs. (4) and (5) it follows that $\frac{d E[UW_{p}]}{d \mu _c}$ and $\frac{d E[UW_{p}]}{d \sigma _c}$ are both positive. In what follows we refer to this complementary analytical feature of convex transfer as **Property 2**. That is, the magnitude of $E[UW_{p}]$ grows in both average $\hbox {CO}_2$ emission growth and its variability.

Therefore, the uncertainty wedge widens in the persistence and volatility of $\hbox {CO}_2$ growth and in the estimated sensitivity of disaster incidence to such growth. Put differently, UW_{p} narrows if either factor is dampened. (Insofar as the global economy successfully adapts to climate change-driven disasters the transfer function’s degree of convexity may weaken. The policy consensus is that there are important synergies between climate risk adaptation (CCA) and disaster risk reduction (DDR) such that CCA and DDR strategies should be unified^19^. Adaptation dynamics lie beyond the scope of the our study.)

**Fig. 2:**
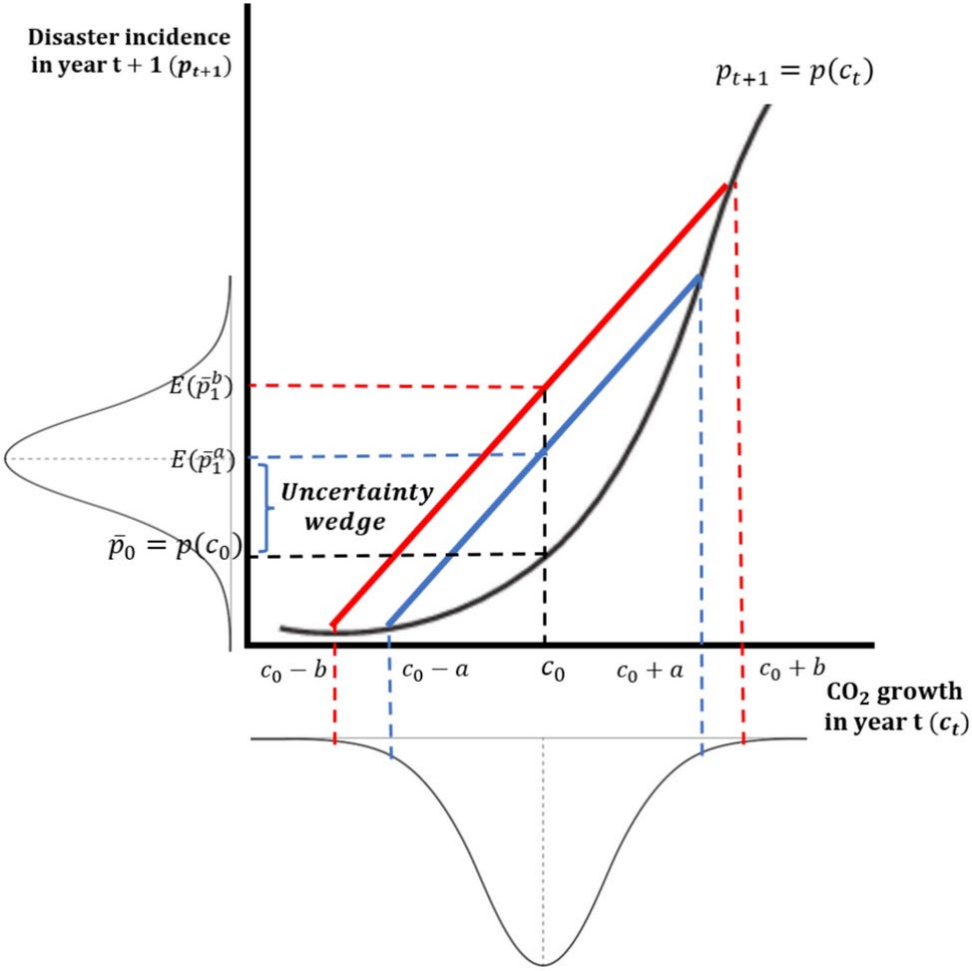
Disaster frequency and $\hbox {CO}_2$ stock variability with logistic transfer.

Figure 2 illustrates such a mean-reducing spread for the logistic function.

Uncertainty is captured by the chord connecting symmetric pair of points $(-a,a)$ and $(-b,b)$ around a given point $c_0$ on the *x*-axis, measuring $\hbox {CO}_2$ growth. We note the chord shifts out with $\hbox {CO}_2$ stock volatility at a given mean. Hence, more stable carbon emissions—for example, moving from *b* to *a*—result in lower expected disaster risk. This relies on the logistic (sigmoid) inflection point being highly positive such that the convex range is to its left; that is the case for all our logit parameter estimates. Indeed, it is easy to check that $\frac{d E[UW_{p}]}{d \sigma _c} < \frac{d E[UW_{p}]}{d \mu _c}$ under a Gaussian pdf and all plausible $\mu _c$ and $\sigma _c$ values.

To summarise, the policy implications of a convex transfer function are the decomposition of total incidence into a certainty-equivalent and an uncertainty wedge component, and the wedge’s monotone response to carbon stock uncertainty. Property 1 suggests that policymakers relying on historical averages as “best guesses” of incidence are likely underestimating disaster events’ expected frequency. Importantly, the underestimate arises not only because the occurrence of extreme events is unprecedented in the observed record^28^ but also because of the positive uncertainty wedge. Property 2 is common in financial risk management as the reverse of a mean-preserving spread^43^, and in monetary policy where it underlies a convex inflation-output trade-off^44^. Climate change-driven disaster risk is part of multiple uncertainties embedded in the carbon cycle: higher output $\rightarrow ^{a}$ higher carbon emissions and concentration $\rightarrow ^{b}$ higher temperature $\rightarrow ^{c}$ climate-related damages $\rightarrow ^{d}$ lower output^29^. Property 2 directly impacts the damages link, circumventing the temperature link of the carbon cycle. Therefore, we should also focus on mitigating the non-negligible uncertainty surrounding emissions.

## Results

### Logit model estimation

Taking logs of Eq. (2) and setting $K=1$ yields the logit transfer function:

6 $$\begin{aligned} logit (p_{it+1}) = \overline{h} + \sum _{j=0}^P h_j c_{it-j} + \gamma \log X_{it} \end{aligned}$$

where logit$(p_{it+1}) = \log (p_{it+1}/(1-p_{it+1}))$ is the log of the odds ratio; $h_j$ are the elasticities measuring the strength of logistic transfer; $c_{it}$ is year-on-year growth in atmospheric $\hbox {CO}_2$ concentration; and $\gamma$ is the elasticity of incidence with respect to the global urban population share $X_{it}$, the only control variable other than carbon stock.

The University of Notre Dame’s GAIN (Global Adaptation Initiative) Index is also included in the robustness checks as an additional control variable in the model to account for differences in vulnerability and resilience across countries (see Appendix B). The index measures a country’s vulnerability to climate change and its readiness to improve resilience, offering a comprehensive assessment that includes social, economic, and infrastructural dimensions. By including the GAIN Index, we capture underlying vulnerabilities and adaptive capacities that influence both the likelihood and impact of disasters. This allows us to control for variations in disaster risk that arise not only from environmental factors but also from social and institutional factors, thereby significantly enhancing the robustness of our model results. (One can also directly control for economic output per capita, population density^27^, and exposure to climatic hazards^19^. However, our study aims to showcase predictive performance and is not intended to provide a comprehensive set of control variables.)

We also introduce country fixed effects that assume countries adopt distinct methods to managing disaster events, and year dummies for time-fixed effects that may capture the potential contribution of key global accords such as the Kyoto Protocol of 1997 and the 2015 Paris Agreement.

Following^31,37,45^, we employ a dynamic panel logit model by incorporating a lagged dependent variable on the right-hand side (RHS). This dynamic specification captures the persistence of climate-related disaster risks. The logistic cumulative distribution function is used as it is particularly suited to modelling extreme events, such as natural disasters. However, dynamic panel models may violate the standard assumptions regarding the error terms, particularly those of independence and lack of serial correlation. This can bias parameter estimates if not appropriately addressed. Serial autocorrelation is indeed identified after plotting the residuals against their lagged values. Therefore, to address this concern, we first tested the model specification by including more lags into the model and then relaxed the assumptions of within-panel correlation and homoskedasticity by using clustered and robust standard errors. We then applied the Generalized Estimating Equations (GEE) model, which is designed to account for autocorrelation and relaxes the assumptions of independence and homoskedasticity in the error terms. Unlike the standard assumption of independent errors, the GEE model allows for a flexible specification of the correlation structure, such as autoregressive structures, which are more suitable when autocorrelation is present in the residuals (see Appendix B).

Also, while two-way fixed effects can control for unobserved heterogeneity to some extent, the assumptions of cross-sectional independence in panel models may not always hold, as highlighted by Refs.^32,46^. Global or regional shocks can introduce common effects across countries. To address this concern, we conducted robustness checks by exploring models with interactive fixed effects (IFE) (IFE helps account for unobserved heterogeneity and common factors that vary across entities and over time, improving the model’s accuracy and robustness^38^. IFE captures cross-sectional dependence and addresses unobserved influences that vary over time, reducing the risk of omitted variable bias. This method thus enhances the model’s fit by allowing for complex interactions, improving both predictive performance and reliability, especially when simple fixed effects are insufficient to capture the full variation in the data^47^.) as presented in Appendix B.

After introducing a composite error term $\epsilon$ yields, the logit specification becomes:

7 $$\begin{aligned} p_{it+1} = \overline{h} + \sum _{j=0}^P h_j c_{it-j} + \gamma \log X_{it} + \delta p_{it} + \delta ^c_i c_i + \delta ^g_t g_t + \epsilon _{it} \end{aligned}$$

where $\delta \ge 0$ measures the persistence of incidence; $\delta ^c_i \ge 0$ and $\delta ^g_t \ge 0$ are the coefficients of the one- and two-way fixed effect dummies, respectively $c_i$ and $g_t$; and $\epsilon _{it}$ satisfies the standard assumptions^14^. The logit parameter estimates with and without fixed effects are in Table 1:

**Table 1:** Disaster incidence 1960–2022: logistic regressions.

Specification control	No FE		1-way FE		2-way FE	
	None	Urban	None	Urban	None	Urban
$$\overline{h}$$	$$-3.607^{***}$$	$$-6.005^{***}$$	$$-5.2$$	$$-9.6$$	$$-5.7$$	$$-6.4$$
$$\hat{h}_0$$ (lag 0)	$$0.497^{***}$$	$$0.485^{***}$$	$$0.504^{***}$$	$$0.456^{***}$$	$$-0.279$$	$$-1.693^{*}$$
$$\hat{h}_1$$ (lag 1)	$$0.347^{***}$$	$$0.305^{***}$$	$$0.360^{***}$$	$$0.277^{***}$$	0.616	$$1.200^{***}$$
$$\hat{h}_2$$ (lag 2)	$$0.423^{***}$$	$$0.358^{***}$$	$$0.432^{***}$$	$$0.326^{***}$$	$$1.242^{***}$$	$$1.719^{***}$$
$$\hat{h}_3$$ (lag 3)	$$0.297^{***}$$	$$0.272^{***}$$	$$0.306^{***}$$	$$0.245^{**}$$	$$0.603^{**}$$	$$1.033^{**}$$
$$\gamma$$	–	$$0.747^{***}$$	–	$$1.276^{***}$$	–	0.204
$$\delta$$	$$0.496^{***}$$	$$0.421^{***}$$	$$0.382^{***}$$	$$0.296^{***}$$	$$0.175^{***}$$	$$0.121^{**}$$
$\overline{\mu }_p$	6.2	6.2	6.2	6.2	6.2	6.2
$d\ln \overline{\mu }_p / d \mu _c$	0.77	0.70	0.79	0.65	0.91	1.11
Pseudo $R^2$	0.128	0.156	0.130	0.144	0.170	0.180
AUROC			0.8616	0.8599	0.8750	0.8722
Obs.	13,298	12,000	13,115	11,820	13,115	11,820

Columns 2–3, 4–5 and 6–7 report maximum likelihood estimates for the logit regression in Eq. (7) estimated for 1960-2022 without fixed effects and with one- and two-way fixed effects, respectively. $$^{***}$$, $$^{**}$$, $$^{*}$$ denote statistical significance at 1, 5 and 10% level, respectively. The estimated coefficients are displayed to 3 d.p. Country-specific ($$\delta ^c_i$$) and time-fixed ($$\delta ^g_t$$) dummy coefficient estimates are not reported to save space; they are available upon request. Certainty-equivalent incidence $$\overline{\mu }_p$$ is calibrated to $${\hat{\mu }}_p \simeq 6.2 \%$$ by choice of $$\overline{h}$$. Response coefficient $$d\ln \overline{\mu }_p / d \mu _c$$ is computed in eq. (8).

Data: EM-DAT is the CRED international hazard and disaster events inventory: www.emdat.be/. The urban population share is available online: data.worldbank.org.

The logit intercepts in *row 3* are computed for each of six reported sets of parameter estimates. For each specification, ${\overline{h}}$ is calibrated such that the certainty-equivalent incidence rate in Eq. (2) matches historical annual disaster incidence from Fig. 1b: $\overline{\mu }_p \rightarrow \hat{p} \approx 6.2$% p.a. The estimated transfer elasticities $\{ \hat{h}_{j=0}^3\}$ measuring the strength of disaster attribution to $\hbox {CO}_2$ growth are significant across the six sets of estimates. When one-way fixed effects are introduced (*columns 4–5*), contemporaneous $\hbox {CO}_2$ growth and its first 3 lags are all significantly positive and the coefficient on urban population share $\gamma$ exceeds one (*column 5*). With two-way effects (*columns 6–7*), lags 1 to 3 remain very significant while the coefficients of contemporaneous carbon growth and urban population share become insignificant; the lagged incidence rate $\delta$ is significant throughout (*row 9*).

**Fig. 3:**
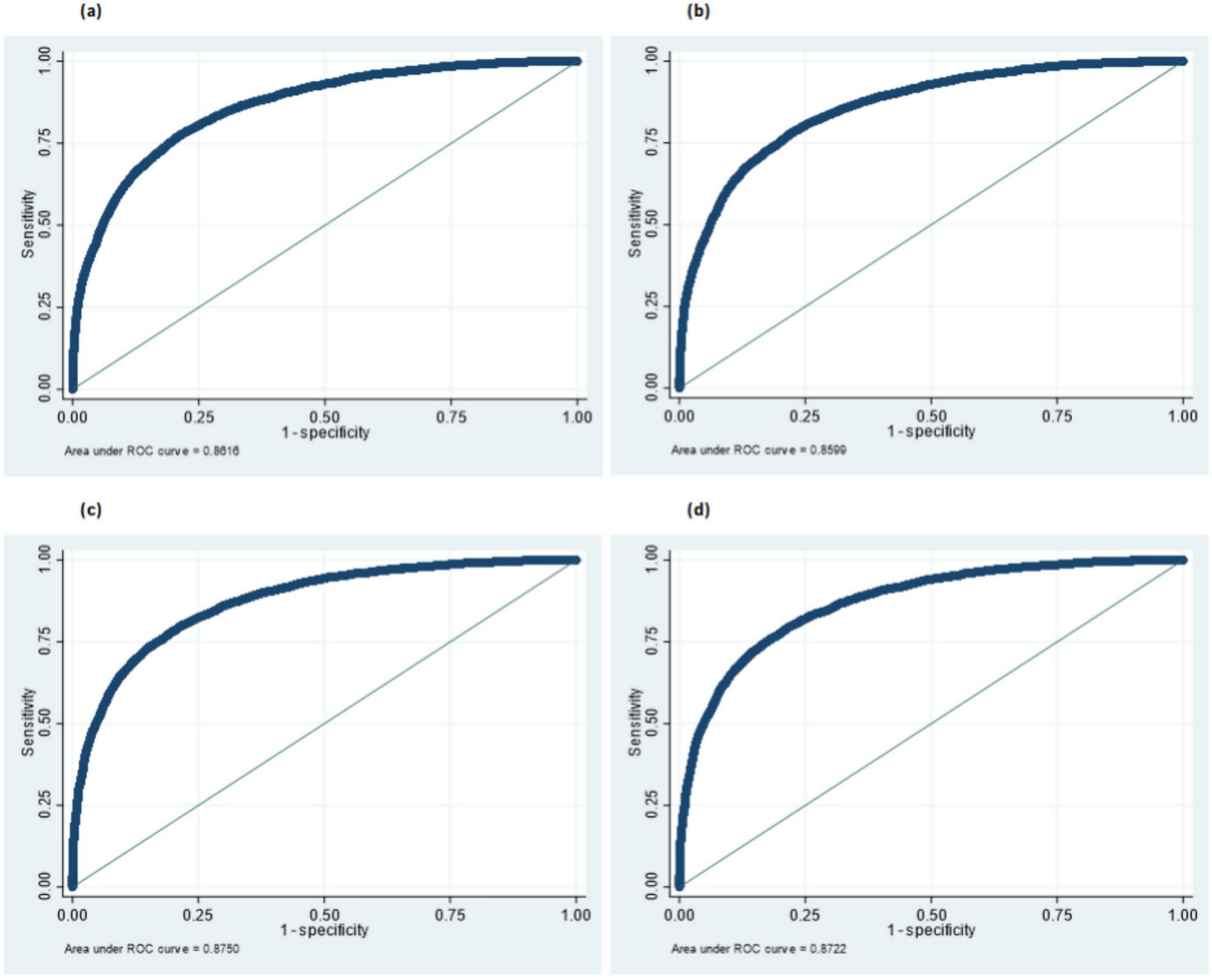
Area under receiver operating characteristic curve (AUROC).

The model has predictive power assessed by the Receiver Operating Characteristic Curve (ROC), the standard method for assessing binary classification ability^14^. (The area under the ROC, denoted AUROC, tests for predictive ability independently of policymakers’ cutoff points. It tests whether the signal distribution generated by the model is significantly different under the disaster and no-disaster states. Thus, it assesses if the signals can be useful in classifing the state outcomes. A perfect classifier would have AUROC = 1.) The AUROC of all 4 specifications with fixed effects are shown in Fig. 3.

The AUROC are in excess of $\approx 0.85$, indicating high classification accuracy.

Further, the cumulative *P*-year percent impact on certainty-equivalent disaster incidence $\overline{\mu }_p$ of varying $\hbox {CO}_2$ concentration by 1 percent controlling for urban population follows from Eq. (2):

8 $$\begin{aligned} \frac{d\ln \overline{\mu }_p}{d \mu _c} \simeq \left( \sum _{j=0}^P h_j \right) \frac{e^{ - \mu _c \sum _{j=0}^P h_j }}{1+e^{ - \mu _c \sum _{j=0}^P h_j }} \end{aligned}$$

The magnitude of $\frac{d\ln \overline{\mu }_p}{d \mu _c}$ is a function of the average value of $\hbox {CO}_2$ growth and its estimated persistence. For our logit regressions $P=3$ and the implied $\frac{d\ln \overline{\mu }_p}{d \mu _c}$ range is [0.65, 1.11] depending on the specification (*row 11*). We now employ this range to project the number of future disaster events. We select the 2015–2022 window to capture the higher recent $\hbox {CO}_2$ emission trend compared to historical emissions when average $\hbox {CO}_2$ growth was $\sim 1.6$% p.a., cf. Fig. 1a. Over this period the cumulative rise in $\hbox {CO}_2$ stock was $\sim 17.5$ ppm, or around $4.4\%$ of base $\sim 401$ ppm level. Hence, the number of disaster events would rise by $\sim [2.86,4.88]\%$ per year for the average country, or [0.0286, 0.048] more disasters. Against the 2002–2021 average of 1.71 disasters per year^23^, this increase implies that under the current trend in $\hbox {CO}_2$ accumulation, for the upper (*lower*) bound of $\frac{d\ln \overline{\mu }_p}{d \mu _c}$ estimates the disaster count would double in 15 (*25*) years to an average 3.42 disasters annually, all else equal. To put these estimates in perspective, Ref.^27^ reports a doubling time of 13 years for hydrological disasters; Ref.^21^ use linear trend extrapolation to project 40% more disaster events by 2030 compared to the average for 1970–2020. (Our estimated range is comparable to those computed by integrated climate models (IAM), see www.iamconsortium.org. A caveat is that as $\frac{d\ln \overline{\mu }_p}{d \mu _c}$ is by definition symmetric, it obscures the likely asymmetric impact incremental that $\hbox {CO}_2$ accumulation may have on disaster incidence due to nonlinear climatic feedbacks, especially if multiple Earth System tipping points are correlated^25^.)

Next, we simulate stochastic disaster incidence by generating $\hbox {CO}_2$ growth paths calibrated to the mean and variance of the 1960–2022 $\hbox {CO}_2$ concentration record. Assuming $c_t$ has finite mean and variance, we first estimate the stationary autoregressive process: (Stationarity has first-order climate policy implications: it implies the expected value of the carbon stock would return to pre-industrial levels ($\sim 280$ ppm) once emissions cease. Although country-level per capita $\hbox {CO}_2$ emissions data at various frequencies and horizons broadly supports mean-reversion^45,48,49^, there is less research on global concentration growth rates. Recent multi-IAM comparisons of emission paths to 2050 also assume stationarity^15^.)

9 $$\begin{aligned} c_t = \rho _0 + \sum ^P_{j=1} \rho _j \text c_{t-j} + \xi _t \, \ \Sigma _{j=1}^P \mid \rho _{j} \mid < 1 \end{aligned}$$

where $c_t$ is the annual mean growth rate of $\hbox {CO}_2$ concentration; and $\xi _t \sim N(0,\sigma _{\xi })$ is Gaussian iid noise. ARMA-ML estimates of $\{\rho _j\}_{j=0}^{P=3}$ are reported in Table 2.

**Table 2:** $\hbox {CO}_2$ stock growth 1960–2022: autoregressions.

Estimation method	ARMA-ML	ARMA-ML	ARMA-ML
Parameter	AR(1)	AR(2)	AR(3)
$$\rho _0$$	$$0.446^{***}$$ (0.000)	$$1.605^{***}$$ (0.000)	$$0.6150^{***}$$ (0.0000)
$$\rho _1$$	$$0.361^{**}$$ (0.010)	$$0.354^{***}$$ (0.006)	$$0.2598^{**}$$ (0.037)
$$\rho _2$$	−	$$0.312^{**}$$ (0.035)	0.2149 (0.184)
$$\rho _3$$	−	−	$$0.3212^{**}$$ (0.018)
Adj. obs.	63	64	64
Mean $c_t$ ($\mu _c$)	0.447	1.624	1.624
S.D. $c_t$ ($\sigma _c$)	0.163	0.680	0.680
AIC/SBIC	$-0.88/-0.74$	1.78/1.91	1.71/1.88
DW	2.07	2.17	2.12
$\hbox {JB}^{res}$ (*p*-val)	(0.31)	(0.47)	(0.24)
Adj. $R^2$	0.14	0.30	0.36
Regr. S.E. ($\sigma _{\xi }$)	0.151	0.568	0.544

The estimated AR(*p*) model is in Eq. (9). *p*-values given in parentheses. Optimal lag order determined using Akaike (AIC) and Schwartz (SBIC) Information Criteria. The intercept is calibrated as $\hat{\rho _0} =(1-\hat{\rho _1}-\hat{\rho _2}-\hat{\rho _3})\mu _c$. The standard error $\sigma _c$ is calibrated using the closed-form solution of the Yule–Walker equations for a strictly stationary AR(3) process^50^.

Data: Annual $\hbox {CO}_2$ growth rates in percent, US National Oceanic and Atmospheric Administration: www.esrl.noaa.gov/gmd/ccgg/trends/.

The estimated 1- and 3-year lag coefficients of the AR(3) model are significant at the 5% level, suggesting strong persistence, while the 2-year lag coefficient is only significant for an AR(2) process. The intercept $\rho _0$ is significant at 1% throughout and the fitted residual distributions do not reject a joint Gaussian pdf. In what follows we adopt the AR(3) estimates for illustrative purposes.

### Baseline calibration

Table 3 presents the calibrated moments of carbon paths generated by applying Gaussian and logistic $\hbox {CO}_2$ growth shocks to Eq. (9):

**Table 3:** Baseline: model-implied $\hbox {CO}_2$ concentration growth.

$c_t = \Delta \log C_t$	1960–2022	Gaussian	Logistic
Obs.	$64 \times 10^3$	$2000 \times 3 \times 10^3$	$2000 \times 3 \times 10^3$
Mean $E[{\tilde{\mu }}_c]$ ($\%$)	[1.46, 1.79]	[1.55, 1.68]	[1.49, 1.76]
$E[{\tilde{\mu }}_c] - {\hat{\mu }}_c$	N/A	$-0.0074$	$-0.0092$
SD $E[{\tilde{\sigma }}_c]$ ($\%$)	[0.60, 0.77]	[0.66, 0.71]	[1.41, 1.55]
$E[{\tilde{\sigma }}_c] - {\hat{\sigma }}_c$	N/A	0.0061	0.7956
Skewness ${\tilde{s}}_c$	$[-0.16,0.55]$	$[-0.13,0.10]$	$[-0.18,0.20]$
Kurtosis ${\tilde{\kappa }}_c$	[1.82, 2.68]	[2.80, 3.22]	[3.34, 4.46]

Column 2: $95\%$ confidence intervals of each statistic in brackets computed for 1000 bootstrap replications of the NOAA $\hbox {CO}_2$ growth series and rounded to 1 d.p. Excess kurtosis is ${\tilde{\kappa }}_p-3$. Data as in Fig. 1a. Columns 3–4: $95\%$ confidence intervals in brackets computed for 2000 runs each $T=2000$ years.

The model-implied mean $E[{\tilde{\mu }}_c]$ is well calibrated to the average growth rate of $\hbox {CO}_2$ concentration for both shock distributions (*row 4*), but only Gaussian shocks match the carbon stock’s historical variability (*row 6*). Logistic pdf shocks also imply excess kurtosis (*row 8*).

Turning to the baseline simulations in Table 4, annual certainty-equivalent disaster incidence is calibrated to $\overline{\mu }_p = 6.2$% throughout. *Columns 2–3* (4–5) report stochastic disaster incidence ($\tilde{p}$) statistics for Gaussian and logistic shocks to $\hbox {CO}_2$ growth conditional on the transfer elasticities estimated with one (two)-way fixed effects.

**Table 4:** Baseline: model-implied disaster incidence.

Incidence	$\hbox {Gaussian}^{1w}$	$\hbox {Logistic}^{1w}$	$\hbox {Gaussian}^{2w}$	$\hbox {Logistic}^{2w}$
$\overline{\mu }_p$ ($\%$)	6.2	6.2	6.2	6.2
Mean $E[{\tilde{\mu }}_p]$ ($\%$)	[6.7,7.8]	[9.5,12.7]	[13.2,15.9]	[24.2,29.3]
$E[UW_{\tilde{p}}]=E[{\tilde{\mu }}_p] - \overline{\mu }_p$ ($\%$)	[0.5,1.6]	[3.3,6.5]	[7.0,9.7]	[18.0,23.1]
SD $E[{\tilde{\sigma }}_p] - {\hat{\sigma }}_p$ ($\%$)	$[-1.8,-0.6]$	$[+5.3,+9.5]$	$[+12.2,+14.9]$	$[+27.7,+30.5]$
RMSE$[{\tilde{p}}, {\tilde{p}}^\mathcal{B}]$	0.013	[0.040, 0.052]	0.102	0.330
Skewness ${\tilde{s}}_p$	[1.2, 2.3]	[2.0, 2.8]	[1.9, 2.3]	[0.9, 1.2]
Kurtosis ${\tilde{\kappa }}_p$	[1.7, 10.0]	[4.2, 9.9]	[2.6, 4.5]	[2.2, 2.9]

$95\%$ confidence intervals in brackets computed for 2000 artificial runs $\{\tilde{p}_{j=1}^{2000}\}$ each $T=2000$ years. Superscripts $^{1w}$ ($^{2w}$) denote parameter estimates using 1 (2)-way fixed effects. RMSE$[{\tilde{p}}, {\tilde{p}}^\mathcal{B}]= \sqrt{ E[(\tilde{p} - {\tilde{p}}^\mathcal{B})^2]}$ computed for model- and beta pdf-implied stochastic incidence realizations $\{{\tilde{p}}\}_{t=1}^T$ and $\{{\tilde{p}}^\mathcal{B}\}_{t=1}^T.$

Note that the incidence means for elasticity coefficients $\{\hat{h}_j\}_{j=0}^3$ estimated with two-way fixed effects are over twice those resulting from the one-way estimates under either shock distribution. This arises because the two-way fixed effect elasticity estimates exceed their one-way counterparts (cf. Table 1), indicating that logistic transfer is relatively more convex. It follows that the uncertainty wedge accounts for $<20 \%$ ($>50 \%$) of total disaster risk for the one (two)-way estimates. In other words, lower (*higher*) elasticity parameter estimates tend to imply lower (*higher*) disaster incidence after controlling for countries’ urban population share. As discussed above, relatively less (*more*) convex logistic transfer in Eq. (7) would indicate weaker (*stronger*) human interference with climate, ceteris paribus. Moreover, the average model-implied volatilities in *row 5* overpredict historical variability of ${\hat{\sigma }}_p=5.6$% p.a. under all specifications except Gaussian shocks and one-way fixed effects, and the root mean square error of model-implied $\tilde{p}$ against trajectories generated by a beta distribution calibrated to the historical record (${\tilde{p}}^\mathcal{B}$) is considerably higher under two-way fixed effects (*row 6*).

Lastly, Table 5 presents simulated disaster risk statistics consistent with the 2.5th (*weak* convexity) and 97.5th (*strong* convexity) percentiles of baseline elasticity point estimates, assuming the mean and variance of $\hbox {CO}_2$ growth are fixed to ${\hat{\mu }}_c$ and ${\hat{\sigma }}_c$. The 3-year cumulative change in certainty-equivalent disaster risk from varying average $\hbox {CO}_2$ stock now ranges from $d\ln \overline{\mu }_p / d \mu _c \sim 0.5$ to $\sim 3$% (*rows 9* and *14*). With one-way fixed effects, expected annual disaster risk in *row 6* exceeds $\overline{\mu }_p$ by upto 3.1% for the 97.5 percentile elasticity estimates (**Property 1**). By contrast, assuming strong convexity the two-way fixed effect parameter estimates imply $E[{\tilde{\mu }}_p]^{2w} \approx 27$% p.a., of which $\sim 75\%$ is the uncertainty wedge (*row 11*). The share of total disaster risk contributed by uncertainty ranges from $\sim 25\%$ with one-way to $>60\%$ with two-way fixed effects.

**Table 5:** Baseline: robustness to convex logistic transfer.

Convexity	Weak		Strong	
CI percentile	2.5	2.5	97.5	97.5
Parameters	$[ \{\hat{h}_j\}_{j=0}^3 ]^{2.5}$	$[ h_{urb}=0]^{2.5}$	$[ \{\hat{h}_j\}_{j=0}^3 ]^{97.5}$	$[ h_{urb}=0]^{97.5}$
$\overline{\mu }_p$ ($\%$ p.a.)	6.2	6.2	6.2	6.2
Mean $E[{\tilde{\mu }}_p]^{1w}$ ($\%$ p.a.)	[6.3, 7.1]	[6.7, 7.8]	[7.1, 8.7]	[7.4, 9.3]
$E[UW_{\tilde{p}}]^{1w}$	[0.1,0.9]	[0.4,1.5]	[0.8,2.4]	[1.2,3.1]
SD $E[{\tilde{\sigma _p}}]^{2w}$	[2.6, 3.1]	[3.6, 4.6]	[5.0, 6.7]	[6.1, 8.3]
$\hbox {RMSE}({\tilde{\mu }}_p,{\tilde{\mu }}_p^B)^{1w}$	0.010	0.013	0.015	0.021
$[{d\ln \overline{\mu }_p / d \mu _c}]^{1w}$	0.47	0.62	0.82	0.95
Mean $E[{\tilde{\mu }}_p]^{2w}$	[16.4, 18.8]	[8.2, 10.1]	[19.2, 26.8]	[14.5, 20.6]
$E[UW_{\tilde{p}}]^{2w}$	[10.2,12.6]	[2.0,4.0]	[13.0,20.6]	[8.3,14.4]
SD $E[{\tilde{\sigma _p}}]^{2w}$	[17.0, 19.3]	[2.4, 4.5]	[22.6, 27.6]	[15.3, 20.6]
$\hbox {RMSE}({\tilde{\mu }}_p,{\tilde{\mu }}_p^B)^{2w}$	0.139	0.033	0.278	0.167
$[{d\ln \overline{\mu }_p / d \mu _c}]^{2w}$	$-0.97$	$-0.17$	3.05	2.26

All entries are in percent p.a. rounded to 1 d.p. The $2.5\%$ and $97.5\%$ confidence point estimates for columns 2–5 are available upon request. For each column the logit intercept $\overline{h}$ is chosen to calibrate $\overline{\mu }_p$. ${\hat{\mu }}_p$ is average disaster incidence from 1960 to 2022. Stochastic measure $E[\tilde{\mu _p}]$ is computed as the average incidence rate realized over 2000 runs of 2000 years each. The expected uncertainty wedge CI, $E[UW_{\tilde{p}}]$, is the difference between the CI for $E[{\tilde{\mu }}_p]$ and $\overline{\mu }_p$. Simulated skewness and kurtosis values are not reported for space constraints; they are available upon request.

In the rest of this paper we maintain the one-way fixed effect elasticity estimates because model-implied expected disaster risk is more robust to the degree of convexity.

## Sensitivity analysis and discussion

**Fig. 4:**
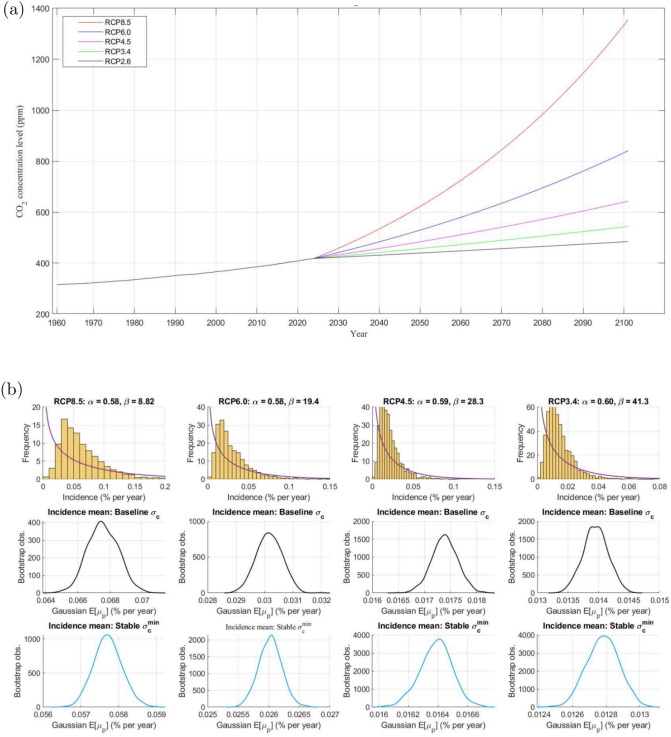
Model projections 2023–2100.

In the previous exercise the unconditional mean and variance of carbon stock were fixed. To explore **Property 2** we now vary both based on IPCC RCP scenarios^1,41^. Following RCP8.5, carbon stock continues to rise through the 21st century, peaking at $\sim 1370$ppm by 2100. At the opposite extreme, $\hbox {CO}_2$ concentrations consistent with RCP2.6 are expected to peak at $\sim 490$ppm sometime before 2100 (2024:09 was $\approx 422$ppm), declining afterwards. (RCP8.5 implies $\mu _c= 1.54$% p.a. between 2023 and 2100, slightly less than the historical mean. It coincides with the baseline calibration of Ref.^35^ as it implies $\hbox {CO}_2$ concentration growth is $\approx 13$ ppm p.a. RCP2.6 assumes full decarbonization: $\mu _c = 0$. Such an ambitious outcome would require massive investment in negative emission technologies and scaled-up $\hbox {CO}_2$ capture.) Figure 4a displays $\hbox {CO}_2$ stock paths consistent with the trend growth rate under each RCP.

Table 6 presents disaster incidence statistics consistent with each pathway’s mean $\hbox {CO}_2$ growth $\mu _c$ (*row 2*) coupled with baseline ${\hat{\sigma }}_c = 0.68 \%$ p.a. (*rows 5–10*), and with stock levels stabilized to $\sigma _c^{\min }=0.30 \%$ p.a., i.e. a stabilized concentration scenario (*rows 11–16*): (We calibrate the $\hbox {CO}_2$ concentration standard deviations for this stabilizing scenario to the multi-model analysis of Ref.^15^. These authors compare the post-2030 and post-2050 emission projections of 7 integrated assessment climate models; see Appendix A.)

**Table 6:** Sensitivity to $\hbox {CO}_2$ stock uncertainty.

2100 horizon	RCP2.6	RCP3.4	RCP4.5	RCP6	RCP8.5
$\mu _c$ ($\%$ p.a.)	0.19	0.34	0.56	0.91	1.54
$\overline{\mu }_p$ ($\%$ p.a.)	1.0	1.2	1.6	2.5	5.6
$\Delta \overline{\mu }_p / \Delta \mu _c$	−	1.5	1.7	2.2	3.5
Mean $E[{\tilde{\mu }}_p \mid {\hat{\sigma }}_c]$	[1.1, 1.3]	[1.3, 1.6]	[1.8, 2.1]	[2.8, 3.3]	[6.0, 7.1]
$E[UW_{\tilde{p}}]$	[0.1,0.3]	[0.1,0.4]	[0.2,0.5]	[0.2,0.7]	[0.4,1.5]
SD $E[{\tilde{\sigma }}_p]$	[0.7, 0.9]	[0.8, 1.1]	[1.1, 1.5]	[1.6, 2.2]	[3.4, 4.4]
Skew ${\tilde{s}}_p$	[1.4, 3.1]	[1.4, 3.2]	[1.4, 3.1]	[1.4, 2.9]	[1.2, 2.4]
Kurt ${\tilde{\kappa }}_p$	[5.8, 22.8]	[5.7, 24.2]	[5.6, 22.6]	[5.4, 20.4]	[4.9, 13.6]
$\Delta E[{\tilde{\mu }}_p] / \Delta \mu _c$ ($\%$)	−	[1.9,3.2]	[2.1,2.9]	[2.5,3.2]	[3.7,4.5]
Mean $E[{\tilde{\mu }}_p \mid \sigma _c^{\min }]$	[1.0, 1.1]	[1.2, 1.3]	[1.6, 1.7]	[2.5, 2.7]	[5.6, 6.0]
$E[UW_{\tilde{p}}]$	[0.0,0.1]	[0.0,0.1]	[0.0,0.1]	[0.0,0.2]	[0.0,0.4]
SD $E[{\tilde{\sigma }}_p]$	0.3	[0.3, 0.4]	[0.5, 0.6]	[0.7, 0.9]	[1.6, 1.9]
Skew ${\tilde{s}}_p$	[0.6, 1.3]	[0.6, 1.3]	[0.6, 1.3]	[0.6, 1.3]	[0.5, 1.1]
Kurt ${\tilde{\kappa }}_p$	[3.3, 6.9]	[3.3, 6.7]	[3.3, 6.6]	[3.2, 6.4]	[3.2, 5.9]
$\Delta E[{\tilde{\mu }}_p] / \Delta \mu _c$ ($\%$)	−	[1.3,1.9]	[1.6,1.9]	[2.1,2.3]	[3.4,3.7]

All entries except rows 4 are $95 \%$ CI rounded to 1 d.p. $\mu _c= \Delta CO_{2t}$ is the average annual $\hbox {CO}_2$ concentration growth from 2023 to 2100 consistent with the 2100 concentration level projected for each pathway. We adopt RCP2.6 as reference for $\Delta E[{\tilde{\mu }}_p] / \Delta \mu _c$ so the specific gradients are undefined. Stochastic $\tilde{p}$ is the average incidence rate over 2000 runs of 2000 years each. Excess kurtosis is ${\tilde{\kappa }}-3$. The baseline elasticity parameter estimates $\{\hat{h}_i\}^3_{i=1}$, $\hat{h}_{urb}$, $\hat{h}_{y_{t-1}}$ are in Table 1, and $\overline{h}$ is chosen to calibrate $\overline{\mu }_p = 6.2 \%$ for each pathway. $E[UW_{\tilde{p}}]$ is computed as in Table 5. The baseline $\hbox {CO}_2$ growth st.dev. is ${\hat{\sigma }}_c = 0.68 \%$ p.a. and the more stable scenario is $\sigma _c^{\min } = 0.30 \%$ p.a.

We make three observations. First, certainty-equivalent disaster incidence $\overline{\mu }_p$ (*row 3*) declines sharply with $\mu _c$, from 5.6% p.a. under RCP8.5 to 1% p.a. under RCP2.6. Mitigated disaster risk reflects lower trend $\hbox {CO}_2$ growth, and the same negative trend is seen for $E[{\tilde{\mu }}_p \mid {\hat{\sigma }}_c]$ in *row 5* (**Property 1**).

Second, while $\overline{\mu }_p$ is only impacted by $\mu _c$, total disaster risk is also sensitive to $\sigma _c$. In particular, $E[{\tilde{\mu }}_p]$ shifts left from $E[{\tilde{\mu }}_p \mid {\hat{\sigma }}_c]$ to $E[{\tilde{\mu }}_p \mid \sigma _c^{\min }]$ (*row 11*). The shift’s magnitude from RCP8.5 to RCP2.6 grows with the ambition of decarbonization targets for 2100. Moreover, for any given $\overline{\mu }_p$, the average uncertainty wedge $E[UW_{\tilde{p}}]$ (*rows 6*, *12*) narrows with more stable emissions (**Property 2**). For example, at the higher end of each confidence interval the expected disaster risk share attributed to uncertainty, $E[UW_{\tilde{p}}] / E[{\tilde{\mu }}_p]$ is about $25\%$ for ${\hat{\sigma }}_c$ but falls to under $10\%$ for $\sigma _c^{\min }$. We also note that average incidence rate volatility drops with $\mu _c$, and more stable carbon emissions result in less fat-tailed and less skewed incidence distributions, all else equal (*rows 14–15*). (By focusing on Gaussian shocks to $\hbox {CO}_2$ stock we have implicitly ruled out catastrophic risk. We conjecture that Properties 1 and 2 extend to extreme value shock probability density functions in the sense of^51^ provided they are symmetric.)

Third, observe that the discrete analogs to response coefficient $\frac{d\ln \overline{\mu }_p}{d \mu _c}$ are $\frac{\Delta \overline{\mu }_p}{\Delta \mu _c}$ for certainty-equivalent disaster risk, and $\frac{\Delta E[{\tilde{\mu }}_p]}{\Delta \mu _c}$ for expected disaster risk. To compute these, we evaluate the response of $\overline{\mu }_p$ and $E[{\tilde{\mu }}_p]$ to a 1% decline in $\mu _c$ from RCP8.5 through to RCP3.4 with base value RCP2.6. The resulting gradients $\frac{\Delta \overline{\mu }_p}{\Delta \mu _c}$ ($\frac{\Delta E[{\tilde{\mu }}_p]}{\Delta \mu _c}$) shown in *row 4* (*10* and *16*) may be viewed as “decarbonisation dividends” in terms of disaster risk reduction. For certainty-equivalent risk they range from 3.5% lower annual disaster likelihood under RCP8.5, to a 1.5% p.a. reduction under RCP3.4. For total expected disaster risk and baseline ${\hat{\sigma }}_c$, the corresponding mitigation is greater even at the $2.5\%$ end of the $\frac{\Delta E[{\tilde{\mu }}_p]}{\Delta \mu _c}$ confidence intervals (3.7 and 1.9%, *row 10*), while for $\sigma _c^{\min }$ the declines are comparable (**Property 1**). The implication is that pursuing decarbonisation policies at decadal frequency pays short-term dividends by reducing annual conditional disaster likelihood, as emphasized by^1^. We also note the discrete gradients exceed the continuous response coefficients reported in Table 1, *row 11*.

Focusing on **Property 2**, expected disaster risk tends to fall with the mean *and* variance of carbon stock growth. Figure 4b sheds light on this key result. The top row displays simulated disaster risk histograms consistent with each RCP trajectory to 2100 from Fig. 4a. It indicates the fitted beta pdf’s $\beta$ parameter rising with the mitigation target; the historical incidence $\beta$ from Fig. 1b stands between RCP8.5 and RCP6.0. The middle (*lower*) rows show smoothed kernel density functions of the expected disaster risk consistent with each pathway for baseline $\hat{\sigma }_c$ ($\sigma _c^{\min }$) and Gaussian $\hbox {CO}_2$ growth shocks. Moving across *columns 1* to *4* highlights that $E[{\tilde{\mu }}_p]$ clearly falls with $\mu _c$. Then, comparing *rows 2* and *3* for a given scenario illustrates the uncertainty wedge narrowing as $\hbox {CO}_2$ stock becomes more stable. For our stylized comparison, expected disaster risk is more sensitive to $\mu _c$ than $\sigma _c$; the incremental decline in total annual disaster risk starting from RCP8.5 is $\sim 3$% across and $<1$% down, shrinking progressively for lower $\mu _c$. Therefore, lowering $\sigma _c$ will complement any reduction in $E[{\tilde{\mu }}_p]$. It follows that RCP1.9, the only pathway limiting global warming to below $1.5^{\circ }\hbox {C}$ (the aspirational goal of the 2015 Paris Agreement) would also benefit from sharply lower disaster risk.

Overall, the results suggest that relying solely on historical averages as best guesses for disaster incidence can lead to an underestimate of their expected stochastic frequency. This key analytical implication of convex transfer may carry significant implications for long-term climate policy as they could lead to more accurate short-term disaster projections (**Property 1**), and insofar as stabilizing $\hbox {CO}_2$ concentrations may result in lower average disaster incidence in addition to decarbonisation policies (**Property 2**). More stable carbon emissions could result from avoiding boom-and-bust cycles, as recommended by Ref.^14^ and briefly witnessed during the COVID-19 pandemic^26^. Implementing such policies will likely require concerted and persistent collective action. In that respect, a key finding of Ref.^33^ is that optimal disaster risk adaptation requires government spending to reduce aggregate tail risk and restore the first-best solution. In turn, adaptation is most effective in mitigating risk under a central-planner equilibrium when households acquire knowledge about disaster risks, such as through public investment in early warning systems, rather than in an environment where no learning occurs. Similarly, Ref.^52^ recommend broadening the scope of carbon pricing schemes, encompassing a wider set of emissions sources and sinks, and extending carbon taxation to the consumption (downstream) stage. We anticipate that such expanded schemes—involving multiple stakeholders and necessitating offsetting public subsidies—have the potential to stabilize $\hbox {CO}_2$ concentration levels.

## Concluding remarks

There is great uncertainty involving the magnitude and pace of climate change. However, there is clear and urgent consensus that human-induced $\hbox {CO}_2$ forcing is a prime driver of disaster events. Against this background, this paper argued that a convex risk transfer function presents policymakers and stakeholders with a strong incentive to collectively pursue more stable carbon emissions. This insight reinforces recent calls for reducing global emissions to protect humanity against irreversible long-term climate tipping risks^5,13^. We estimated a dynamic panel logit model linking the global disaster and atmospheric $\hbox {CO}_2$ records from 1960 to 2022, and assessed the risk reduction potential of five IPCC decarbonisation scenarios, corresponding to sharply different climate policies up to 2100. The logit model’s AUROC scores were statistically significant and exceeded 0.85, indicating the model’s utility as a decision aid in one-year-ahead disaster prediction. Further, we established that uncertainty regarding the growth of $\hbox {CO}_2$ concentration introduces a wedge between stochastic and certainty-equivalent disaster risks. This arises because disaster incidence is a convex function of $\hbox {CO}_2$ growth, and its magnitude increases with the elasticity of logistic transfer and with the persistence of carbon concentration. Although most of our findings remain robust after controlling for the GAIN index and interactive fixed effects (IFEs), calibrating a transfer function that comprehensively accounts for socio- and techno-economic factors and exploring more heterogeneous effects across different regions would be a natural extension of the model’s application.

## Supplementary Information

Supplementary Information 1.

Supplementary Information 2.

Supplementary Information 3.

## Data Availability

The datasets used and/or analysed during the current study are included as supplementary files. Please contact Saite Lu (sl590@cam.ac.uk) if you have further questions.
