# Supplementary material for: Stabilising CO2 concentration as a channel for global disaster risk mitigation
Source: Sci Rep. 2024 Nov 24;14:29120. doi: 10.1038/s41598-024-79437-5 (PMC11586419; doi:10.1038/s41598-024-79437-5)
Supplement: Supplementary file 1 — Supplementary Information 1. [file 41598_2024_79437_MOESM1_ESM.pdf]

Appendix A.

A1. Disaster incidence: Simulations

$T = 2000$ Gaussian i.i.d. CO_2 growth shocks are drawn from a Gaussian $N(0, \sigma_\xi)$ pdf, where σ_ξ is the AR(P) regression standard error reported in Table 2, and from a logistic pdf with $\mu = 1.608$, $\sigma = 0.401$ fitted to the 1960-2022 observations. These shocks are applied to the estimated persistence parameters of the AR(P) specification ($\{\rho_j\}_{j=0}^{P}$). Each artificial shock sequence is initialized by setting the first P values to zero, and each sequence generates a CO_2 growth path of T annual increments. $H = 2000$ such shock runs are generated and their moments calibrated to the historical mean μ_c and standard deviation σ_c of CO_2 growth. The calibration quality is < 0.005. The resulting array of $T \times H$ artificial CO_2 years is fed to eq. (7) based on the estimated logit transfer coefficients of each regression specification. Fixing the urban population share control to its historical mean $\{\mu_{K=1}\}$ yields H stochastic incidence vectors of size $T \times 1$ years each. Eq. (2) then produces one certainty-equivalent incidence rate $\overline{\mu}_p$ for each estimated parameter set. The first 4 simulated disaster incidence moments are computed for each run, and 97.5 percent confidence intervals are generated across all runs.

A2. Representative Concentration Pathway(RCP): Assumptions and specification

Different RCP compiled by the IPCC are ranked according to their projected level of radiative forcing in 2100. They include RCP2.6 and RCP8.5, the best and worst-case climate scenarios for 2100 in terms of end-horizon carbon concentration, as well as intermediate cases. The original four Representative Concentration Pathways (RCP) were developed since 2007 to coordinate climate modeling

assumptions ahead of the IPCC Fifth Assessment Report (*AR5*) in 2014 (Van Vuuren et al., 2011). There are now six RCP spanning a range of radiative forcing values (in W/m^2), from 1.9 to 8.5 W/m^2. The Earth System's energy imbalance is produced by total greenhouse gas (GHG) atmospheric concentrations in the year 2100 employed in integrated climate models (IAM) and converted to CO_2 equivalent concentrations.

RCP-implied time paths are representative in that each represents a wider range of scenarios, and each includes a CO_2 concentration level (in parts per million, ppm) reached in 2100. We have selected all except RCP1.9, the only pathway limiting global warming to below 1.5°C above pre-industrial levels. A range of five Shared Socioeconomic Pathways (SSP) is also available and was employed to help produce the IPCC Sixth Assessment Report (*AR6*) in 2021 (Masson-Delmotte et al., 2021). However, SSP are not suited for our purposes as they are conditional on specific projected socio-economic outcomes, including future agriculture yields, population dynamics, energy use etc. As we only condition on future CO_2 concentration paths we fix control variables to their unconditional mean, hence RCP are appropriate.

Compared with present global atmospheric concentration levels and the number of years to 2100, preset end-horizon concentrations allow computing average CO_2 growth rates. Specifically, given the 2023 CO_2 concentration of $C_{2023} \simeq 418$ppm, an RCP trajectory with terminal CO_2 concentration level C_{2100} is consistent with annual average growth rate x, where:

$$\log(CO2_{2023}) + (2100 - 2023) * \log(1 + x) = \log(C_{2100})$$

It follows that $x^{8.5} \simeq 1.54$ percent p.a. is consistent with RCP8.5; $x^{6.0} \simeq 0.91$

percent p.a. with RCP6.0; $x^{4.5} \simeq 0.56$ percent p.a. with RCP4; $x^{3.4} \simeq 0.34$ percent p.a. with RCP3.4; and $x^{2.6} \simeq 0.19$ percent p.a. with RCP2.6.

We employ the 2100-horizon CO_2 emission projections of seven established IAM analysed by Sognnaes et al. (2021) as a proxy for CO_2 stock levels: E3ME, FortyTwo, GCAM, GEMINI, ICES, MUSE and TIAM. The seven diverse models' key characteristics are summarized in Table 2 of Sognnaes et al. (2021). That study compared model projections under the assumption of continuing rates of emission-intensity reductions (emissions per GDP unit), as well as increasing carbon prices in line with per capita economic growth. There is no direct conversion between CO_2 emissions and concentrations, and the impact nonlinear climate change feedback effects, including abrupt tipping phenomena, is highly uncertain. As discussed in section 1, it is only very recently that the geophysical and geochemical tipping mechanism have started being systematically incorporated in generating long horizon CO_2 stock projections; see Schleussne et al. (2024) and Moller et al. (2024). With that caveat, up to first order the atmospheric CO_2 increase of 1 tCO_2 emitted today is 0.45 tCO2. The standard deviations, in percent p.a., of each IAM projected emission trajectory are 0.21 (GCAM), 0.26 (Forty Two, E3ME), 0.27 (GEMINI), 0.30 (ICES, TIAM) and 0.32 (MUSE). Therefore, $\sigma_c^{\min} \approx 0.30$ percent p.a. appears a good approximation for the stabilised concentration scenario over the 2023-2100 period investigated in Section 4.

Appendix B. Robustness Checks

TABLE B1. Results from Different Lag Specifications and the Probit Model: 1960-2022

Model	1-way FE		2-way FE		2-way FE	
Specification	**(1)**	**(2)**	**(3)**	**(4)**	**(5)**	**(6)**
$\overline{h}$	–	–	–	–	-1.884^{***}	-2.428^{***}
$\hat{h}_0$ (lag 0)	0.399^{***}	0.364^{***}	-0.521	0.192	-0.129	-1.035^{*}
$\hat{h}_1$ (lag 1)	0.239^{***}	0.173^{***}	1.296^{***}	0.160	0.316	0.687^{***}
$\hat{h}_2$ (lag 2)	0.296^{***}	0.245^{***}	1.156^{***}	0.358^{**}	0.711^{***}	1.012^{***}
$\hat{h}_3$ (lag 3)	0.235^{***}	0.210^{***}	0.321	0.309^{**}	0.343^{***}	0.616^{**}
$\hat{h}_4$ (lag 4)	0.189^{***}	0.178^{***}	-0.326	0.146^{***}	–	–
$\hat{h}_5$ (lag 5)	–	0.264^{***}	–	0.512^{***}	–	–
$Urban$	1.152^{***}	1.019^{***}	–	0.142	–	0.133
δ	0.279^{***}	0.262^{***}	0.169^{***}	0.111^{*}	0.132^{***}	0.096^{**}
Logit	Y	Y	Y	Y	–	–
Probit	–	–	–	–	Y	Y
Obs.	11,623	11,426	12,900	11,426	13,115	11,820

Note: Columns (1)-(4) report maximum likelihood estimates for the panel logit regression models with extended lag specifications for the period 1960-2022. Columns (5) and (6) present estimates from panel probit models, where the standard errors are adjusted to account for intragroup correlation and heteroskedasticity. ***, **, and * denote statistical significance at the 1%, 5%, and 10% levels, respectively. The estimated coefficients are displayed to three decimal places.

Data: Data on hazard and disaster events are sourced from EM-DAT, CRED. Urban population share data is obtained from the World Bank.

TABLE B2. Logistic Regressions with Relaxed Assumptions: 1960-2022

Model	2-way FE		2-way FE		2-way FE	(winsorised)
Specification	**(1)**	**(2)**	**(3)**	**(4)**	**(5)**	**(6)**
$\overline{h}$	-3.473^{***}	-4.365^{***}	-3.510^{***}	-4.393^{***}	-4.285^{***}	-4.224^{***}
$\hat{h}_0$ (lag 0)	-0.282	-1.732^{*}	-0.319	-1.754^{*}	-1.764^{*}	-0.757
$\hat{h}_1$ (lag 1)	0.626	1.225^{***}	0.658^{*}	1.240^{***}	1.252^{***}	1.042^{***}
$\hat{h}_2$ (lag 2)	1.267^{***}	1.756^{***}	1.323^{***}	1.777^{***}	1.791^{***}	1.436^{***}
$\hat{h}_3$ (lag 3)	0.615^{***}	1.055^{**}	0.661^{***}	1.072^{**}	1.082^{**}	0.833^{**}
$Urban$	–	0.208	–	0.214	0.161	0.041
δ	0.179^{**}	0.124	-0.074	0.045	0.038	0.026
Logit	Y	Y	–	–	–	–
GEE	–	–	Y	Y	Y	Y
Obs.	13,115	11,820	13,115	11,820	11,820	11,820

Note: ***, **, * denote statistical significance at 1, 5 and 10 percent level, respectively. Columns (1) and (2) report the estimated results from the logit model with two-way fixed effects, where the standard errors are adjusted for intragroup correlation and heteroskedasticity. Columns (3) and (4) present the results from the Generalized Estimating Equations (GEE) model, which accounts for autocorrelation and relaxes assumptions about the correlation structure of the error terms. Autoregressive patterns are specified in these models. Columns (5) and (6) replicate the results from (3) and (4), but with the explanatory variables winsorised at the 1st and 99th percentiles, and the 5th and 95th percentiles, respectively.

Data: Same as in Table B1.

TABLE B3. Box-Tidwell Test

Model	1-way FE		2-way FE		2-way FE	
Specification	**(1)**	**Interaction**	**(2)**	**Interaction**	**(3)**	**(4)**
$\overline{h}$	-4.811^{**}	–	-25.596^{***}	–	-0.452	-1.563^{*}
$\hat{h}_0$ (lag 0)	0.896^{***}	-0.289	-8.175	4.371	0.085	-2.647
$\hat{h}_1$ (lag 1)	-0.074	0.197	26.797^{***}	-13.961^{***}	0.506	1.873^{***}
$\hat{h}_2$ (lag 2)	0.374	-0.050	-4.424	2.686	1.795^{***}	2.707^{***}
$\hat{h}_3$ (lag 3)	0.214	0.014	2.335	-0.985	0.708	1.520
$Urban$	0.945	0.087	3.285^{**}	-1.513^{*}	–	0.214
δ	0.777^{***}	–	0.019	–	-0.073	0.045
GEE	Y		Y		Y	Y
Log form	–	–	–	–	Y	Y
Obs.	11,820	–	11,820	–	13,115	11,820

Note: ***, **, * denote statistical significance at 1, 5 and 10 percent level, respectively. Regressions (1) and (2) report the results of the Box-Tidwell test, where the Interaction columns provide the coefficients and their statistical significance for the interaction terms, used to test for non-linearity in the specified models. Regressions (3) and (4) present the results of models with log-transformed independent variables to address potential non-linearity issues. All models are estimated with one-way and two-way fixed effects, as indicated.

Data: Same as in Table B1.

TABLE B4. Logistic Regressions with Additional Controls and Interactive Fixed Effects (IFE): 1960-2022

Model	1-way FE	2-way FE	2-way FE	2-way FE
Specification	**(1)**	**(2)**	**(3)**	**(4)**
$\overline{h}$	-0.175	-0.880	-0.507^{***}	0.218
$\hat{h}_0$ (lag 0)	0.182	0.251	0.302^{***}	0.144^{**}
$\hat{h}_1$ (lag 1)	-0.042	0.074	0.304^{***}	0.108^{*}
$\hat{h}_2$ (lag 2)	0.147^{**}	0.273^{***}	0.215	0.103^{*}
$\hat{h}_3$ (lag 3)	0.237^{***}	0.176^{*}	0.362^{**}	0.102^{**}
$GAIN$	-0.032	0.038^{*}	–	-1.090
$Urban$	–	–	1.447^{***}	0.929
δ	0.378^{***}	0.131	-1.956^{***}	-1.874^{***}
GEE	Y	Y	Y	Y
IFE (PC1-3)	–	–	Y	Y
Obs.	4,564	4,564	12,000	4,914

Note: ***, **, * denote statistical significance at 1, 5 and 10 percent level, respectively. The GAIN index is added as an additional control in Columns (1) and (2). Columns (3) and (4) incorporates Interactive Fixed Effects (IFE) by applying the principal components approach and retaining the residuals of all the independent variables in the regression, which help account for unobserved heterogeneity and cross-sectional dependence.

Data: GAIN index is produced by the Notre Dame Global Adaptation Initiative. Other data are the same as in Table B1.
